# Understanding the psychosocial experiences of adults with mild-moderate hearing loss: An application of Leventhal’s self-regulatory model

**DOI:** 10.3109/14992027.2015.1117663

**Published:** 2016-01-12

**Authors:** Eithne Heffernan, Neil S. Coulson, Helen Henshaw, Johanna G. Barry, Melanie A Ferguson

**Affiliations:** 1 National Institute of Health Research, Nottingham Hearing Biomedical Research Unit, Nottingham, UK; 2 Otology and Hearing Group, Division of Clinical Neuroscience, School of Medicine, University of Nottingham, Nottingham, UK; 3 Division of Rehabilitation and Ageing, School of Medicine, University of Nottingham, Nottingham, UK; 4 Medical Research Council Institute of Hearing Research, Nottingham, UK; 5 Nottingham University Hospitals NHS Trust, Nottingham, UK

**Keywords:** Hearing loss, Leventhal’s self-regulatory model, common sense model, illness representations, psychosocial impact, qualitative research

## Abstract

**Objective:**

This study explored the psychosocial experiences of adults with hearing loss using the self-regulatory model as a theoretical framework. The primary components of the model, namely cognitive representations, emotional representations, and coping responses, were examined.

**Design:**

Individual semi-structured interviews were conducted. The data were analysed using an established thematic analysis procedure.

**Study sample:**

Twenty-five adults with mild-moderate hearing loss from the UK and nine hearing healthcare professionals from the UK, USA, and Canada were recruited via maximum variation sampling.

**Results:**

Cognitive representations: Most participants described their hearing loss as having negative connotations and consequences, although they were not particularly concerned about the progression or controllability/curability of the condition. Opinions differed regarding the benefits of understanding the causes of one’s hearing loss in detail. Emotional representations: negative emotions dominated, although some experienced positive emotions or muted emotions. Coping responses: engaged coping (e.g. hearing aids, communication tactics) and disengaged coping (e.g. withdrawal from situations, withdrawal within situations): both had perceived advantages and disadvantages.

**Conclusions:**

This novel application of the self-regulatory model demonstrates that it can be used to capture the key psychosocial experiences (i.e. perceptions, emotions, and coping responses) of adults with mild-moderate hearing loss within a single, unifying framework.

Hearing loss is a widespread condition, affecting approximately 328 million adults globally (World Health Organization, 2014). Not only is hearing loss pervasive, it is also a long-term condition that can have substantial psychosocial consequences. Perhaps the most substantial of these consequences are communication difficulties and social isolation (Strawbridge et al, 2000; Kramer et al, 2002; Dalton et al, 2003; Pronk et al, 2013). In addition, people with hearing loss can experience stigmatization, as the condition has various negative connotations, including old age, incompetence, cognitive impairment, and social impairment (Southall et al, 2010; Wallhagen, 2010). In the labour market, individuals with hearing loss are more likely to have lower wages and higher unemployment rates (Hogan et al, 2009; Jung & Bhattacharyya, 2012). There is also some evidence that hearing loss is related to depression, cognitive decline, and dementia (Kramer et al, 2002; Acar et al, 2011; Lin et al, 2011; Lin, 2011; Boi et al, 2012).

While it is clear that hearing loss can have a considerable psychosocial impact, current understanding of this impact would be greatly enhanced if it were underpinned by an established theoretical framework. This could enable disparate strands of research on the subject to be drawn together to form a cohesive narrative. It could also provide new insights on the psychosocial aspects of hearing loss. Recently, hearing researchers have turned to the discipline of health psychology to identify models that have been successfully applied to other health conditions and that could improve the understanding of the behaviours and experiences of individuals with hearing loss (Manchaiah, 2012). For example, the transtheoretical model and the health belief model have been used in investigations of hearing health behaviours, such as help-seeking and hearing-aid use (Laplante-Lévesque et al, 2013, 2015; Saunders et al, 2013). To investigate the psychosocial impact of hearing loss, the present study utilised the self-regulatory model (SRM), also known as the common sense model (Leventhal et al., 1980). This model has its origins in health psychology research from the late 1960s and early 1970s, which examined whether the sensation of fear and the perception of a health threat were related to relatively acute health behaviours, such as tetanus vaccination and smoking reduction. The model was extended to chronic health conditions by examining how individuals’ emotional reactions to and beliefs about their condition influence their selection, performance, and maintenance of coping responses (Leventhal et al, 1997; Hale et al, 2007). In the decades since its development, the SRM has been applied to numerous long-term conditions but has rarely been used in hearing research.

The SRM (Figure 1) posits that a stimulus, such as a symptom or diagnosis, prompts individuals to develop cognitive and emotional representations of their condition. Cognitive representations are lay beliefs about the condition stemming from personal knowledge and experiences, information from the media, and information from significant others, whereas emotional representations are subjective reactions to the condition, such as anxiety or fear (Hagger & Orbell, 2003). Cognitive representations have five main components (Petrie & Weinman, 1997; Hale et al, 2007): (1) **identity**, or beliefs about the symptoms and labels associated with the condition, (2) **causal beliefs**, or beliefs about the factors that led to the development of the condition, (3) **timeline**, or beliefs about the duration of the condition, (4) **controllability/curability**, or beliefs about the extent to which the condition can be controlled, treated or cured and (5) **consequences**, or beliefs about the short and long term effects of the condition.

The SRM proposes that cognitive and emotional representations influence the selection of coping responses, which in turn influence health outcomes (Hagger & Orbell, 2003). Coping responses are actions taken to solve problems posed by the condition or actions taken to regulate feelings stirred by the condition. A coping response can be multifunctional, as it can both alleviate physical symptoms and emotional distress (Leventhal et al, 1997; Hale et al, 2007). Once selected, individuals monitor and evaluate their coping responses. These evaluations determine whether individuals amend or maintain their coping responses and also whether they amend or maintain their original cognitive and emotional representations. This process is known as the feedback loop (Johnston, 1997; Hagger & Orbell, 2003; Hale et al, 2007). For example, the evaluation of a coping response as unsuccessful may prompt individuals to perceive their condition as uncontrollable and to select an alternative coping response (Leventhal et al, 1997). Both coping responses and representations can directly influence health outcomes. For example, the representation of a condition as controllable/curable has been associated with improved psychological well-being and social functioning (Hagger & Orbell, 2003).

The SRM was selected for this study as it is an established framework that has been successfully applied to numerous long-term conditions, including diabetes, psoriasis, and epilepsy (Petrie & Weinman, 1997; Hagger & Orbell, 2003). Within hearing research, it has been applied to auditory processing disorder, sometimes known as King-Kopetzky syndrome (Pryce et al, 2010). There is strong support for the tenets of the model, with many studies confirming that representations are predictive of both health behaviours, particularly adherence, and health outcomes (Leventhal et al, 1992; Sharpe & Curran, 2006). In addition, various studies have demonstrated that it is an appropriate model for the exploration of the psychosocial experiences of patients (e.g. Barsevick et al, 2001; Lingler et al, 2006). The SRM is also an advance on other health psychology models, as it recognises the important influence of emotion on health behaviours and it considers how individuals choose and appraise coping responses (Leventhal et al, 1997; Sharpe & Curran, 2006).

The aim of this study was to explore the psychosocial experiences of adults with mild-moderate hearing loss using the SRM as a theoretical framework. Specifically, the study explored the cognitive and emotional representations of individuals with hearing loss, as well as their perceptions of their coping responses. The results will be used to inform the development of a questionnaire that measures the psychosocial impact of hearing loss. The study adopted a qualitative approach, as qualitative research is an essential element of questionnaire development (Brod et al, 2009; Lasch et al, 2010). In addition, the qualitative approach is the optimum approach for obtaining rich insights into individuals’ experiences, beliefs, desires, values, and motivations (Ives & Damery, 2014). Recently, Knudsen et al (2012) called for greater use of qualitative methods in hearing research to deepen our understanding of the experiences and perceptions of individuals with hearing loss and to potentially uncover information that may have been overlooked by quantitative research. The specific qualitative data collection method chosen for this study is one of the most well-established: the individual semi-structured interview. The advantage of this technique over alternative techniques, such as focus groups, is that it is particularly suited to the exploration of sensitive and personal topics (Brinkmann, 2014).

## Method

### Sampling and recruitment

Two groups of participants were recruited: (1) adults with mild-moderate hearing loss, as defined as having a mean hearing threshold between 20–70 dB HL in the better ear averaged across 0.25–4 kHz or a unilateral hearing loss (British Society of Audiology, 2011), and (2) hearing healthcare professionals. The purpose of obtaining the views of different stakeholders, known as triangulation, was to enhance the rigour of the study (Yardley, 2008). Adults with hearing loss were recruited through the Nottingham Hearing Biomedical Research Unit (BRU) participant database via email or post. Hearing healthcare professionals were recruited from the authors’ professional network via email. Maximum variation sampling was carried out, such that sampling continued until participants with diverse characteristics and experiences were recruited (Patton, 1990). All participants were offered a small inconvenience allowance, as well as travel expenses.

### Participants

Twenty-five adults with hearing loss (14 men) living in the UK participated in the study (Table 1). The mean age was 68.76 years (SD = 16.45, range = 20–91 years). The mean pure-tone hearing threshold was 40.84 dB HL (SD = 14.52, range = 18–69 dB HL) in the better ear, averaged across 0.25–4 kHz. All owned hearing aids, with 22 wearing them regularly (i.e. at least several times per week). In addition, nine hearing healthcare professionals (two men) living in the UK, USA, and Canada participated in the study (Table 2). They included audiologists, hearing therapists and academics.

### Procedure

The research was approved by the East Midlands NHS Research Ethics Committee and the Nottingham University Hospitals NHS Trust. A pilot study was conducted with two adults with hearing loss and two audiologists. The adults with hearing loss each participated in a pilot interview and hearing assessment. As this did not result in any notable changes to the interview schedule, the data of the two adults with hearing loss were included in the analysis. The audiologists reviewed the interview schedule for the hearing healthcare professionals and suggested revisions. The interview schedules are available as supplementary material in the online version of the journal. Please find this material with the direct link to the article at: http://dx.doi.org/10.3109/14992027.2015.1117663. The schedules were flexible due to the semi-structured design of the interviews, though their core content remained the same across each interview. Written informed consent was obtained from each participant prior to their interview. The first author conducted all of the interviews, each of which typically lasted 60 minutes. Thirty interviews were conducted face-to-face in a quiet room in the Nottingham Hearing BRU. Four hearing healthcare professionals who were not located in Nottingham were interviewed via online video call. Each interview was audio-recorded and subsequently transcribed verbatim.

For the adults with hearing loss, a hearing assessment was conducted to ensure that they had mild-moderate hearing loss. Otoscopy was performed prior to the measurement of pure-tone air conduction thresholds (0.25–8 kHz) for each ear and pure-tone bone conduction thresholds (0.5–2 kHz) in accordance with the British Society of Audiology (2011) procedure. In addition, all adults with hearing loss completed the Glasgow Hearing Aid Benefit Profile or GHABP (Gatehouse, 1999) by interview (Table 1). This provided a validated measure of subjective hearing disability (i.e. activity limitations) and handicap (i.e. participation restrictions).

### Data analysis

Thematic analysis was performed by the first author in accordance with the procedure outlined by Braun and Clarke (2006). QSR International’s NVivo 10 software supported the analysis. The data of the two participant groups were analysed together, such that themes common to both groups were sought. The thematic analysis was deductive (Braun & Clarke, 2006), as it was informed by the SRM. The analysis began with an in-depth review of the interview recordings and transcripts. Subsequently, the entire dataset was coded, including extracts that appeared to be unrelated to psychosocial experiences or the SRM, so that nothing of importance was overlooked. A process of combining or redefining the codes led to the generation of initial themes. Overarching themes stemmed from the model (e.g. cognitive representations), whilst sub-themes either stemmed from the model (e.g. identity) or were devised by the first author (e.g. muted emotions). Disconfirming case analysis, or examining participants and extracts that differ from the themes identified, was performed to strengthen the rigour of the analysis. The rigour was further bolstered by a coding comparison (Yardley, 2008). Specifically, a researcher, who was not otherwise involved in the study, independently coded a representative sample of six of the transcripts and formulated potential themes. A comparison of the two analyses indicated that there were no substantial discrepancies, suggesting that the interpretation of the data was not limited to the perspective of the first author. The themes were refined and defined through re-analysis of the data and discussions amongst the co-authors.

## Results

The results are discussed in terms of the primary components of the SRM. An identification code has been assigned to each adult with hearing loss (e.g. AHL1) and each hearing healthcare professional (e.g. HHP1).

### Theme 1: Cognitive Representations of Hearing Loss

#### Identity

Individuals with hearing loss tended to see hearing loss ‘symptoms’ in terms of activity limitations and participation restrictions, such as difficulties with watching television, using the telephone, and conversing with the others. Many associated hearing loss with various negative labels, one of the most common of which was being seen as ‘old’. While those who saw ageing as a natural process were not especially concerned by this label, others found it upsetting. AHL17 said: “*I want people to see me as me; not me with a hearing aid or me with a [walking] stick*…*I want them to see me as I was*”. Hearing loss was also commonly associated with looking ‘stupid’ or ‘silly’. HHP1 (hearing therapist/academic) said: “*There is this fear of appearing stupid, which perhaps doesn’t happen with other disabilities*”. Some found that hearing loss was associated with a lack of competence and authority. AHL16 stated: “*I have always been*…*ever so efficient and capable and, you know, running things and organising things but because of my hearing, all that has gone*”. Some found that hearing loss was related to being seen as ‘unfriendly’ and ‘difficult’. This is because communication difficulties (e.g. not replying when addressed) and communication tactics (e.g. asking people to speak more clearly) can be confused with rudeness by those who have little awareness of hearing loss. AHL20, who initially concealed her hearing loss, said:

“*I actually made an effort not to talk to people*…*So when I would be around people I would probably have my head stuck in a book. So I probably came across as quite ignorant and unapproachable*”. She added: “*after I got my hearing aids somebody did actually say to me that they had been worried because I had been so quiet*…*and they thought*…*I was a loner*”.

#### Causal beliefs

When asked about the causes of their condition, individuals with hearing loss most commonly cited ageing and/or noise exposure. However, few had a clear understanding of the causes of their hearing loss and some had little interest in learning more. Indeed, three hearing healthcare professionals suggested that patients often receive more information about hearing loss than they need. HHP2 (audiologist/academic) said: “*Many people feel that patients should be able to*…*rattle off their audiogram and many patients don’t particularly care*”. Similarly, HHP3 (academic) said: “A*udiologists tend to give way too much information*…*[Patients] want to know if there’s a fix and how can they stop it getting worse. They don’t need to know all of the miniscule details*”. Such a lack of interest in a detailed understanding of hearing loss may be beneficial, with HHP4 (audiologist/academic) noting that some patients might become fixated on examining the causes of their hearing loss, rather than accepting the condition and learning to cope with it.

Contrasting with this perspective, some individuals with hearing loss and clinicians proposed that understanding the causes and nature of hearing loss could help people to accept the condition and to understand why they need audiological rehabilitation. AHL24 said: “*Reading more about it and trying to understand more about it is my way of coping with it*”. HHP5 (hearing therapist) said: “*Like with anything in life*…*if we have an explanation; if we have a foundation, we are able to then get to grips with it*”. Ultimately, several of the professionals expressed the view that it is best to tailor the information given to patients based on their individual preferences.

#### Timeline

Most individuals with hearing loss were not especially concerned about the progression of their condition. Many had come to accept that their hearing would continue to decline and were determined to carry on regardless. AHL1 said: “*It is a gradual deterioration. So I don’t have any anger*, *frustration*…*I passively accept that this is how it will be and just get on with doing what I can*”. There were a small number who reported worrying about further decline. While some overcame this worry with time, others continued to feel anxious, particularly if they believed that their hearing loss could become unmanageable in the future. AHL24 began to learn sign language in case her hearing deteriorated: “*I will be 68 in twenty years’ time*…*will I have lost my hearing by then or will it be just a little lower than what it is now?*…*It is upsetting*…*because I think, well, how will I communicate with people?*”

#### Controllability/curability

individuals with hearing loss can vary greatly in terms of whether or not they feel in control of their hearing loss. HHP1 (hearing therapist/academic) said:

“*I have probably seen people at all points on the spectrum from*…*people who are very much*…*“I have got a hearing loss but it doesn’t stop me doing anything”*…*to people at the other end, who are like, “I just don’t know what I am going to do*…*my whole life has fallen apart,” and then there is*…*everything in between*”.

Most individuals with hearing loss in this study believed that they could not control or cure their condition, yet this did not appear to hinder their coping. Instead, they had come to accept their hearing loss and were motived to use hearing aids and other coping strategies. AHL23 said:

“*It is outside of my control. There is nothing so certain as that. The only way I can control it is by putting hearing aids in and adjusting them*…*You have got to realise that nothing, nothing is going to replace your hearing*…*What you can do is find something which will enhance what you have got*…*if you are not prepared to accept it then I am sorry; you have got a bit of a rotten life*”.

A small number of individuals with hearing loss hoped that a cure could yet be developed. AHL15 said: “*I wish you could give me back my hearing*…*so I don’t need to wear hearing aids at all, but I just have to accept it really*…*until you invent something that will help. I expect eventually there will be*”. Also, some initially believed that hearing aids would restore normal hearing. HHP5 (hearing therapist) said: “*The expectation is that a hearing aid fixes your hearing and I don’t know whether that is the fault of [the] explanation or*…*whether we, as humans, kind of hope for it to fix things*…*but [it] often sets people up for a fall*”.

#### Consequences

The individuals with hearing loss reported a small number of positive consequences of hearing loss, such as being able to ignore unpleasant sounds and disturbances (e.g. loud music, car alarms) and developing a greater awareness of hearing loss and other disabilities. However, for most, any silver lining was outweighed by the negative consequences of hearing loss. These included the negative impact hearing loss can have on identity, with some even feeling stigmatised by the condition. Another consequence is the experience of various negative emotions, discussed in greater detail under the emotional representations theme.

The most substantial consequences of hearing loss reported by the participants were activity limitations and participation restrictions. In particular, individuals with hearing loss struggle to communicate with others, especially in noisy environments, on the telephone or in group conversations. They can also find communicating with strangers demanding and intimidating, as strangers have unfamiliar communication styles and may lack awareness of hearing loss. AHL14, who had sudden-onset hearing loss, said: “*I just wanted to be on my own and [with] people that I knew*…*I was frightened to meet new people because you don’t know how they speak*”. Some find formal interactions difficult, such as interactions with doctors, managers and colleagues. In particular, they may feel uncomfortable about disclosing their hearing loss and asking for support in a formal context. HHP1 (hearing therapist/academic) explained: “S*omething I have had so many times is ‘I couldn’t hear the doctor in the appointment and I didn’t want to say’*…*So then there is a*…*worry*…*with people thinking: ‘Well actually, what did he actually tell me?’*”

Hearing loss can also considerably affect the relationships between individuals with hearing loss and their communication partners. Individuals with hearing loss can find it difficult to take part in family gatherings and to converse with family members, particularly grandchildren. AHL9 said: “*I am with the family and they are talking and I feel as though I am not in the same world*”. Hearing loss can also place a strain on romantic relationships. Some individuals with hearing loss find that they have fewer joint social activities with their partner, fewer enjoyable conversations with their partner, and greater friction in their relationship. For example, AHL3 described how her boyfriend was irritated by having to repeat himself: “*He just gets annoyed at me and doesn’t bother telling me what he has just said*…*We have lived together for just under two [years] and he still can’t cope with it*”. Friendships are also affected, particularly as friends often meet in challenging listening environments, such as pubs and restaurants. AHL5 said: “*Where I have difficulty is sitting in a gathering with friends and the conversation is flowing*…*I am perhaps more taciturn than I might otherwise be*”.

Hearing loss can also restrict participation in various social, leisure and community activities. AHL16 said: “*I am part of the prayer ministry team*…*a couple of weeks ago I said: “I am really going to have to come off it’*…*because I can’t do it. I cannot hear what people want prayer for*”. She went on to explain the significance of having to sacrifice this activity: *“it is*…*something else that is stripped away*…*it is not just your hearing that you have lost; it is a lot of other things you have lost as well*”. In addition, hearing loss can negatively affect participation in educational activities, especially listening in lectures and contributing to group discussions. It can also affect numerous aspects of work life, including taking part in meetings, participating in training courses, and building relationships with colleagues.

### Theme 2: Emotional Representations of Hearing Loss

#### Negative emotions

Most individuals with hearing loss reported negative emotional representations of hearing loss. Initial emotional reactions included disbelief, anger, and fear. HHP6 (audiologist) explained: “Y*ou do go through the stages of grief and anger and disappointment and ‘why me?’*…*before you can come to anything else*”. The individuals with hearing loss often overcame these initial emotions, as they accepted and adjusted to their hearing loss. However, many found that they still experienced negative emotions in daily life because of their hearing loss. In particular, many felt frustrated and irritated, primarily due to communication difficulties and the limitations of hearing aids. HHP1 (hearing therapist/academic) suggested that irritation, though a relatively mild emotion, can take a toll on wellbeing when it becomes an everyday presence. Also, many reported feeling embarrassed by having a hearing loss, by wearing hearing aids, and by having communication difficulties. Some even conceal their hearing loss from others. Another common sensation was loneliness, or isolation, largely due to communication difficulties and participation restrictions. AHL4 said: “*If you go to weddings or christenings*…*all these people around you are having a good time and you are*…*isolated because you are not fully part of the group*”. Also some experienced worry, especially in relation to missing important sounds and information.

#### Positive emotions

There were a small number of reports of positive emotional representations of hearing loss. HHP7 (audiologist) suggested that many patients in clinic are relieved to have an explanation for their hearing difficulties and are grateful for opportunity to receive help. AHL3 was “*pleased*” to be diagnosed with hearing loss as a teenager: “*I always loved the idea of having hearing aids*…*when you are sixteen-seventeen, you want something special about you*…*I also liked the idea that there was a reason for why I was having trouble.*” However, such a positive emotional response was largely unique to AHL3 and, unfortunately, her emotions become less positive as she realised that hearing aids would not ‘fix’ her hearing loss.

#### Muted emotions

The results indicated that some individuals with gradual-onset hearing loss experience a relatively calm emotional reaction to the condition, possibly because they have time to accept and adjust to hearing loss or because they do not regard it as a serious condition. AHL7 described realising that his hearing was declining: “*I don’t know [that] I had many feelings about it*…*I well understand it is natural and ageing*”. HHP8 (audiologist/academic) suggested that some only become emotional when they reflect on how hearing loss has restricted their participation: “*The emotional response will start when they think about*…*participation in the particular situation*…*Until then, they’re*…*fine, but when you talk about a particular situation*…*they get emotionally a bit worked up*”.

### Theme 3: Coping Responses

The individuals with hearing loss displayed two main coping responses. The first, disengaged coping, means avoiding addressing hearing loss, such as by denying or ignoring it or by withdrawing from social situations. The second, engaged coping, means taking action in order to manage hearing loss, such as using hearing aids and communication tactics.

#### Disengaged coping

Two primary forms of disengaged coping emerged. The first, **withdrawal from situations**, refers to avoiding being physically present in challenging situations, such as social gatherings. HHP5 (hearing therapist) said: “*People self-isolate quite a lot, I think. As situations become harder and harder to manage, the much easier option is to not do it*”. AHL20 said: “*I was missing out on life*…*I was probably isolating myself from social situations*…*it was just too much effort to try and hear what people were saying*”. Rather than entirely withdraw from all social situations, individuals with hearing loss tended to participate in some situations and not others. Some found that they could no longer partake in the social activities they most valued. For example, AHL14 left her ideal job in the police force, though she had permission to stay in the role: “*I did give up my police [job] because I knew I couldn’t put myself or a colleague in danger by*…*having this disability. So my childhood dream had to come to an end*”. AHL9, who regarded family as “*The most important thing*”, was no longer able to babysit her great-grandchildren: “*I feel I am too old to babysit for them because I couldn’t hear what they were saying*…*it is depressing really*”.

The second form of disengaged coping, **withdrawal within situations**, refers to being physically present in social situations but being a passive rather than an active presence in those situations. According to HHP1 (hearing therapist/academic) this may be the most prevalent form of withdrawal: “*They don’t go or they withdraw within the situation, which is perhaps more common*…*they say that: ‘I went along but I couldn’t really follow the conversation. So I was just*…*nodding and smiling.’*” Group conversations were the main situation in which individuals with hearing loss reported ‘switching-off’, as it can become too difficult and fatiguing to attempt to contribute to the discussion. AHL16 said: “*You are there but you are not there*”. She added: “*you just sit there like a fool and everything is going on around you*”. AHL8 said: “*You*…*say to yourself: ‘Is it important that I need to get involved in this conversation?’*…*you do sometimes adopt an isolationist attitude and say, ‘Well I am not going to pick up everything that is going on. So why bother?’*” Some rely on communication partners to follow the conversation on their behalf. Some use ‘bluffing’ by pretending that they can follow the conversation.

Disengaged coping can lead individuals with hearing loss to respond inappropriately to questions, to miss important information, to feel isolated in social situations and to become less socially active. Nevertheless, there are those who prefer this approach, as it allows them to avoid the stress and fatigue associated with socialising and the embarrassment of displaying one’s hearing difficulties to others.

#### Engaged coping

Many individuals with hearing loss were determined to continue with their daily lives, despite their hearing loss. AHL5 said: “*You either concentrate on the negative side of it*…*Or you say,* ‘*Well, that is how it is. Now let*’*s get on with it*’…*Which sounds terribly pompous and flag-waving but*…*you effectively do that*”. The majority regularly wore hearing aids and found them to be beneficial. AHL20 said: “*They are not just hearing aids any more. They are part of me*”. Nevertheless, the participants reported that hearing aids have their limitations. Some felt that hearing aids can be uncomfortable, unattractive, and associated with ageing. Some mentioned that they gain little benefit from hearing aids in noisy environments, such as social gatherings, which means that their participation remains somewhat restricted.

Many reported successfully using communication tactics, although these tactics were seen as inappropriate in certain circumstances. Specifically, communication tactics, such as asking for repetition, can spoil group conversations, especially when a joke or story is being told. AHL6 said: “Y*ou are conscious of the fact [that] if you say anything, you are breaking into the story or you are breaking into the conversation. So you don’t want to do that. So you keep quiet and you don’t hear what they are saying*”. Some felt that communication tactics are ineffective when interacting with people who lack awareness of or sympathy towards hearing loss. In addition, some felt that using communication tactics, such as asking people to speak clearly, can result in them being perceived as demanding, annoying, or stupid. Communication tactics also require assertiveness, which does not come naturally to everyone.

## Discussion

This study aimed to explore the psychosocial experiences of adults with mild-moderate hearing loss using the self-regulatory model (SRM) as an underpinning theoretical framework. The meaningfulness of the model’s primary components (i.e. cognitive representations, emotional representations, and coping responses) to the psychosocial experiences of individuals with hearing loss was examined. The findings will be used to inform the development of a new measure of the psychosocial impact of hearing loss.

### Cognitive representations of hearing loss

In terms of identity, hearing loss was found to have various negative connotations, including old age, unintelligence, and unfriendliness. This aligns with previous investigations of the stigmatisation of hearing loss and its impact on one’s sense of identity (Espmark & Scherman, 2003; Southall et al, 2010; Wallhagen, 2010). In terms of causal beliefs, there was a divergence of opinion amongst the participants as regards the benefits of developing a detailed understanding of the nature and causes of hearing loss. Ultimately, the professionals recommended tailoring the provision of clinical information to each individual patient. Indeed, such patient-centred approaches are now at the forefront of auditory rehabilitation (Grenness et al, 2014a, 2014b; Ferguson et al, in press).

In terms of timeline, most individuals with hearing loss were not particularly concerned about the progression of their condition. Also, most believed that hearing loss is not controllable or curable. Despite this belief, the majority regularly wore hearing aids. This contrasts with a meta-analysis of SRM studies, which showed that perceived controllability/curability is positively associated with active, problem-focused coping (Hagger & Orbell, 2003). As mild-moderate hearing loss is typically irreversible and progressive, it may be better for individuals with hearing loss to accept that they have a long-term condition with which they must learn to live, rather than hope for an improvement to their hearing. Also, it is possible that individuals with hearing loss can perceive the condition itself to be uncontrollable and incurable, but nevertheless believe that its symptoms or consequences can be more effectively managed through hearing aids and other coping strategies. This study indicates that perceiving that hearing loss has low controllability/curability is not necessarily detrimental to engagement in auditory rehabilitation.

This study showed that hearing loss was perceived as having primarily negative consequences. The most substantial of these consequences were activity limitations and participation restrictions, which confirms findings from previous research (Dalton et al, 2003; Helvik et al, 2006). In particular, individuals with hearing loss often experienced communication difficulties, strained relationships with communication partners, and difficulties taking part in social, leisure, community, and professional activities. Hearing loss was perceived as having some positive consequences, though these tended to be outweighed by the negative consequences of the condition. This supports previous studies that demonstrated that hearing loss has some positive outcomes, including stronger relationships with communication partners, reduced disturbance from undesired sounds, affinity with other individuals with hearing loss, and improved concentration, creativity, and self-reliance (Kerr & Stephens, 1997; Stephens & Kerr, 2003; Yorgason et al, 2007).

### Emotional representations of hearing loss

Individuals with hearing loss had primarily negative emotional responses to the condition, including frustration, embarrassment and loneliness. The findings suggested that emotional responses can shift over time, reflecting the long-term nature of hearing loss. Emotional representations of hearing loss warrant further attention, as the SRM posits that they can be an important influence on individuals’ management of their health conditions (Leventhal et al, 1997). Also, a recent investigation of audiology appointments found that emotional concerns expressed by patients were often overlooked by their audiologist. The authors recommended that audiologists attend to these emotional concerns to improve the therapeutic relationship and to increase the likelihood of the patient adhering to rehabilitation (Ekberg et al, 2014).

### Coping responses

There were two primary coping responses: disengaged coping, or avoiding addressing one’s hearing loss, and engaged coping, or taking action to manage one’s hearing loss. This corresponds to some extent with Hallberg and Carlsson’s (1991) qualitative study, which proposed that people with hearing loss use two main coping strategies: avoiding the social scene (e.g. pretending to understand others, avoiding social situations) and controlling the social scene (e.g. making the best of social situations, asking people to repeat themselves). The present study has introduced the concepts of withdrawal from situations and withdrawal within situations as the two primary forms of disengaged coping. Withdrawal from situations entails avoiding being physically present in social situations (e.g. declining a party invitation), while withdrawal within situations entails being physically present in social situations without actively participating in those situations (e.g. sitting quietly whilst others converse). This suggests that individuals with hearing loss who attend many social events could appear, on the surface, to have a high degree of social functioning, yet they could feel quite isolated and dissatisfied during those events. In addition, individuals with hearing loss could take part in a wide range of social activities without taking part in the activities they most value, such as babysitting their grandchild. Therefore, successful social functioning for individuals with hearing loss is not necessarily attending many social events, but rather being able to fully participate in and enjoy the social situations that they deem important. This relates to the proposal that social isolation has both an objective component; social network size, and a subjective component; perceived loneliness (Hawthorne, 2008; Weinstein et al, 2015).

Despite the disadvantages of disengaged coping, particularly social isolation, it can allow individuals with hearing loss to avoid embarrassment, fatigue, and stress in social situations. Similarly, engaged coping was perceived to have both advantages and disadvantages. Most of the individuals with hearing loss found hearing aids helpful, yet acknowledged that they have various drawbacks, including reduced benefit in noisy environments. Many reported using communication tactics, though they were seen as ineffective in certain situations, such as when they obstruct group conversations or when communication partners are unsympathetic. The finding that both disengaged coping and engaged coping have perceived benefits and drawbacks is supported by previous research. Gomez and Madey (2001) found that individuals with hearing loss can perceive both ‘adaptive’ coping (e.g. asking for repetition) and ‘maladaptive’ coping (e.g. pretending to understand) to be effective, even though the latter does not facilitate communication. It is possible that some feel that ‘maladaptive’ coping enables them to avoid embarrassment and social rejection (Jaworski & Stephens, 1998). As such, categorising coping strategies as either ‘adaptive’ or ‘maladaptive’ may be too simplistic, as a strategy’s appropriateness can depend on the specific individual and the specific situation (Andersson & Willebrand, 2003). For example, individuals with hearing loss can prefer to use communication tactics with familiar, rather than unfamiliar, communication partners (Tye-Murray et al, 1992; Caissie et al, 1998). It is important that clinicians consider these complexities, especially the potential limitations of communication tactics, when counselling patients.

## Limitations

While this study supports the merits of applying the SRM to hearing loss, the model is not without its limitations. McAndrew et al (2008) argued that while there is an abundance of healthcare studies describing the model, there have been few attempts to utilize it in the development of clinical interventions. The model has also been critiqued for omitting personal and contextual factors (Leventhal et al, 1997; Hale et al, 2007). Other frameworks, particularly the International Classification of Functioning, Disability and Health (World Health Organization, 2001), regard such factors as important influences on activity, participation, and physical functioning. To overcome this limitation, the present study used open-ended questions to explore the SRM, which Diefenbach and Leventhal (1996) argue enables personal and contextual factors to be captured.

The potential limitations of this study must also be addressed. Firstly, the participants with hearing loss were recruited from a database of individuals who were willing to take part in research investigating their hearing difficulties. This means that they may be more likely to be accepting of their hearing loss and to be relatively socially active and thus they may not be representative of all individuals with hearing loss. To counteract this, professionals were also interviewed to provide a broader perspective based on their experiences with a range of patients in clinic. Secondly, the study used a deductive, rather than an inductive, thematic analysis approach, which arguably increases the risk of overlooking important aspects of the psychosocial experiences of individuals with hearing loss where they do not fit within the framework of the SRM. While this risk cannot be denied, it is a concern for all thematic analysis approaches since no researcher is entirely free from preconceptions, including their pre-existing knowledge of the relevant literature and theories (Malterud, 2001; Braun & Clarke, 2006).

## Conclusion

This study used the SRM to explore the psychosocial experiences of adults with mild-moderate hearing loss. While the psychosocial impact of hearing loss has been examined in previous studies, the application of health psychology theory to this subject is still in its infancy. This exploratory, qualitative study could provide a foundation for future applications of the SRM to hearing loss, including quantitative investigations of the components of the model or explorations of the relevance of the model to other populations with hearing loss, such as those with severe to profound hearing loss. The findings support existing research, by confirming that hearing loss is perceived as having primarily negative consequences and primarily negative connotations. Additionally, the study uncovered various novel findings relating to emotional representations, including positive, negative, and muted emotional reactions to hearing loss, and relating to cognitive representations of the timeline, controllability/curability, and causes of hearing loss. The study also showed that both engaged and disengaged coping have perceived benefits and limitations and that disengaged coping can take the form of either physically withdrawing from social situations or mentally withdrawing within social situations. These findings demonstrate the power of the SRM to provide unique and rich insights into the psychosocial experiences of individuals with hearing loss. In particular, the SRM enables key elements of the psychosocial experiences of individuals with hearing loss to be captured within a single, unifying framework.

## Figures and Tables

**Figure 1:**
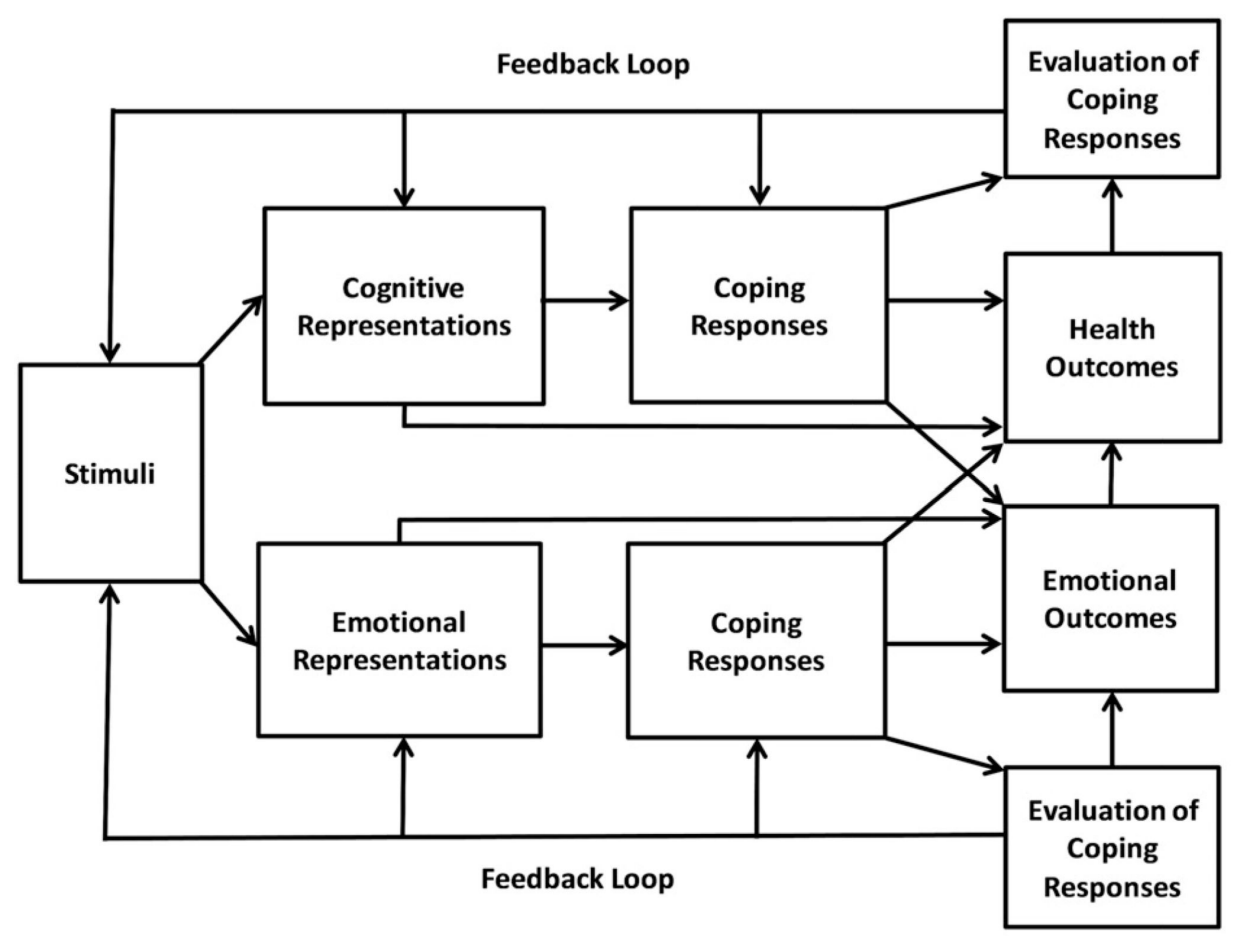
Leventhal’s (1980) Self-regulatory model. This figure has been adapted from Hagger and Orbell (2003).

**Table 1:** Demographic information of the adults with hearing loss.


**Gender**	**N**
Male	14
Female	11
**Age**	**Years**
Mean	68.76
SD	16.45
Median	72
Range	20–91
**Better ear pure-tone average**	**dB HL**
Mean	40.84
SD	14.52
Median	36
Range	18–69
**Hearing loss onset**	**N**
Gradual	21
Sudden	2
Congenital	1
Unknown	1
**Employment status**	**N**
Retired	18
Employed	5
Not Employed	1
In Education	1
**GHABP scores**	**Mean Percentage (N = 25)**
Hearing Disability (Activity Limitations)	38.13 (SD = 20.67, Range = 6.25–81.25)
Hearing Handicap (Participation Restrictions)	39.09 (SD = 27.31, Range = 0–93.75)
Hearing Aid Use	82.32 (SD = 33.30, Range = 0–100)
Hearing Aid Benefit	57.98 (SD = 27.29, Range = 6.25–100)
Hearing Aid Satisfaction	56.70 (SD = 20.79, Range = 6.25–81.25)


**Table 2:** Demographic information of the hearing healthcare professionals.


**Gender**	**N**
Male	2
Female	7
**Location**	**N**
UK	6
USA	2
Canada	1
**Profession**	**N**
Audiologist	6
Hearing Therapist	2
Academic	1
**Current occupation**	**N**
Audiologist	3
Hearing Therapist	1
Academic	5


## Abbreviations

SRM: Self-regulatory model
BRU: Biomedical Research Unit
GHABP: Glasgow Hearing Aid Benefit Profile
AHL: Adult with hearing loss
HHP: Hearing healthcare professional
